# Long-Term Valproate Treatment Increases Brain Neuropeptide Y Expression and Decreases Seizure Expression in a Genetic Rat Model of Absence Epilepsy

**DOI:** 10.1371/journal.pone.0073505

**Published:** 2013-09-09

**Authors:** Johanna Elms, Kim L. Powell, Leena van Raay, Stefanie Dedeurwaerdere, Terence J. O’Brien, Margaret J. Morris

**Affiliations:** 1 Pharmacology, School of Medical Sciences, University of New South Wales, Sydney, Australia; 2 The Department of Medicine, The University of Melbourne, Royal Melbourne Hospital, Melbourne, Australia; 3 Department of Translational Neurosciences, University of Antwerp, Belgium; Kaohsiung Chang Gung Memorial Hospital, Taiwan

## Abstract

The mechanisms by which valproate, one of the most widely prescribed anti-epileptic drugs, suppresses seizures have not been fully elucidated but may involve up-regulation of neuropeptide Y (NPY). We investigated the effects of valproate treatment in Genetic Absence Epilepsy Rats from Strasbourg (GAERS) on brain NPY mRNA expression and seizure control. GAERS were administered either valproate (42 mg.kg^−1^ hr^−1^) or saline continuously for 5 days. Electroencephalograms were recorded for 24 hrs on treatment days 1, 3 and 5 and the percentage of time spent in seizure activity was analysed. NPY mRNA expression was measured in different brain regions using qPCR. Valproate treatment suppressed seizures by 80% in GAERS (p<0.05) and increased NPY mRNA expression in the thalamus (p<0.05) compared to saline treatment. These results demonstrate that long-term valproate treatment results in an upregulation of thalamic expression of NPY implicating this as a potential contributor to the mechanism by which valproate suppresses absence seizures.

## Introduction

Valproate is one of most commonly prescribed anti-epileptic drugs and is effective in a broad range of seizure types. Valproate is generally regarded as the first-line anti-epileptic drug for patients with generalised epilepsy syndromes [1]. However, its precise mechanism(s) of action is still not fully understood. Recently it has been proposed that the up-regulation of neuropeptide Y (NPY) in the brain may play a role in the anti-epileptic action of valproate based on the observation that healthy rats chronically treated with valproate for four days showed increased expression of NPY in the nucleus reticularis thalami (nRT) and the hippocampus [2].

A variety of mechanisms have been implicated as being involved in mediating valproate’s pharmacological effects. These include increased GABA synthesis and release resulting in increased GABAergic transmission [3], [4], [5], decreases excitatory synaptic activity through the modulation of postsynaptic non-NMDA receptors [6] and blockade of voltage-dependent sodium channels [7], [8]. *In vitro* experiments show that valproate has an early effect to inhibit cellular excitability, which is exerted from the extracellular side of the neuronal membrane, and a more delayed effect resulting from intracellular actions [9], [10]. These data indicate that there are both immediate and long-term actions of valproate on cellular excitability.

NPY is a 36 amino acid peptide that is abundantly expressed in GABAergic interneurons of the mammalian central nervous system with highest expression seen in the cerebral cortex, dentate hilus, striatum, the reticular nucleus of the thalamus and the arcuate nucleus of the hypothalamus [11], [12] and is known to be an endogenous suppressor of seizure activity [13]. NPY signals through identified Y1, Y2, Y4, and Y5 receptors that couple to G-proteins, inhibiting adenylate cyclase and thus decreasing intracellular calcium levels [14], [15]. Multiple receptors have been implicated in mediating NPY’s seizure suppression action, including Y1, Y2 and Y5 subtypes [16], [17], [18].

There is substantial evidence for an important role of NPY in the regulation of epileptic seizures. NPY immunoreactivity and Y2 receptor binding is increased in the hippocampus in patients with temporal lobe epilepsy [19] and NPY expression is increased in the rodent brain after recurrent seizures induced chemically or electrically [20], [21], [22], [23], [24], [25]. Additionally, exogenously administered NPY has been shown to suppress seizure activity in cortical and hippocampal slices in vitro [26], [27], [28], in experimental models of epilepsy, including the Genetic Absence Epilepsy Rats from Strasbourg (GAERS) model of genetic generalised epilepsy [13], [18], [29], after kainic acid-induced seizures [30] and chronic infusions of NPY delays amygdala kindling epileptogenesis [31]. Further evidence for the importance of NPY in epilepsy has come from NPY knockout and transgenic mice. Mutant mice lacking NPY have an increased susceptibility to spontaneous and pharmacologically induced seizures [32] and are unable to terminate limbic motor seizures [33]. On the other hand, transgenic rats overexpressing NPY have reduced susceptibility to induced seizures [34].

This study tested the hypothesis that increases in NPY expression in the thalamus or cortex, the primary brain regions involved in the oscillatory neuronal network activity that underlies absence seizures, occur with long-term valproate administration in an animal model of genetic generalized epilepsy. We also examined whether NPY expression was increased in the arcuate nucleus of the hypothalamus, and the relationship of this to food intake.

## Methods

### Ethics Statement

All procedures on rats were approved by The University of Melbourne Animal Ethics Committee (Ethics Number 0705408) and conformed to National Health and Medical Research Council Guidelines for the ethical use of animals in scientific research. All efforts were made to minimize stress and the number of animals necessary to produce reliable data.

### Animals

GAERS are an inbred Wistar rat strain that begin to manifest absence-like seizures after 4–6 weeks of age accompanied by 5–8 Hz spike-and-wave discharges (SWDs) recorded on the EEG [35]. The seizures in GAERS exhibit a similar pharmacological response to anti-epileptic drugs as for human absence epilepsy, being suppressed by valproate and ethosuximide and aggravated by carbamazepine [36], [37], [38]. For this study GAERS aged between 4–6 months were obtained from the breeding colonies at the Royal Melbourne Hospital and Ludwig Institute for Cancer Research Melbourne, Australia. After surgery, animals were placed in individual cages and handled daily to acclimatize them to handling. Animals were fed a normal diet of tap water and rat chow. All procedures were approved by the Melbourne Health Animal Ethics committee and followed the Australian Code of Practice for the care and use of animals for scientific purposes.

### Surgery

Rats were anaesthetised with isoflurane (0.3 L.min^−1^ oxygen and 0.2 L.min^−1^ air) and placed in a stereotaxic frame (Stoelting, USA). Six electrodes and two screws located ventrally on the skull were implanted above the dura. The electrodes were inserted into a 9-pin ABS plug (GS09PLG-220, Grinder Scientific) and dental cement (Vertex TM, B.V Netherlands) used to fix ABS plug and electrodes to skull. Saline (0.9% NaCl, Astra Pharmaceuticals, North Ryde, NSW, Australia) was injected subcutaneously to prevent dehydration and carprofen (5 mg.kg^−1^, Pfizer, Australia) was given intraperitoneally (i.p.) for pain relief. A one week recovery period was allowed before any further procedures were performed.

### Jugular Vein Cannula Implantation

Our laboratory has devised an infusion cable that allows for prolonged simultaneous EEG acquisition and intravenous infusion [38], [39]. This involves implantation of a jugular vein cannula (PVC ID = 0.8 mm, OD = 1.2 mm, Microtube Extrusions Pty Ltd, Australia) filled with heparinised saline (20 IU.ml^−1^ Heparin Sodium DBL, Hameln Pharmaceuticals GmbH, Germany) and tunnelled through the back of the neck to the muscles surrounding the jugular vein. A small incision was made in the vein and the tubing was inserted 2.5 cm until it reached the superior vena cava of the heart. After checking the position of the cannula it was fixed to the surrounding muscles. The ABS plug with cannula attached, was then connected to the cable.

### Valproate Infusion

Post cannulation rats were immediately infused with heparinised saline (1 ml.hr^−1^) using a multi-syringe infusion/withdrawal pump (KDS 230 Walker Scientific, Australia). The cable was connected to a fluid swivel that was attached via a swivel mount to a commutator and then to a syringe infusion/withdrawal pump [39]. Patency of the cannula was monitored once daily by withdrawal of blood into the cannula and food intake was recorded at 11 am each day.

Rats remained on the infusion set up for 8 days in total. All rats (n = 17) were given heparinised saline as described above for 3 days and on the 4^th^ day rats were weighed and assigned randomly to either 42 mg.kg^−1^.hr^−1^ valproate (Sigma, USA) (n = 9) a dose that is known to suppress seizures [38], [40] or saline (n = 8). After 5 days of treatment (day 8) rats were euthanized with an overdose of sodium pentobarbital (1 ml/kg i.p.;Virbac, Australia) followed by rapid extraction of the brain.

### EEG Recordings and Analysis

The electrodes were attached to wires by gold crimp pins that connected to a computer running Compumedics™ EEG acquisition software (Melbourne, Australia). EEGs were recorded over a 24 hr period and analysed using Spike-and-Wave Finder™ software (provider Van Den Broek, Nijmegen University, the Netherlands). An observer, who was blinded to the nature of the treatment, verified quantification of seizure expression. The following criteria were used to define a seizure: SWD burst of amplitude of more than three times baseline, a frequency of 6 to 12 Hz, and duration of longer than 0.5 s. EEG data from the baseline period at the end of saline run in, and on days 1, 3 and 5 of valproate or vehicle infusion were analysed. The percentage of time spent in seizure activity, number of seizures per hour and seizure duration was calculated in the 24 hour period for each animal and valproate treatment was compared to saline treatment at each time point.

### Blood Sampling and Brain Dissection

At the completion of the study rats were euthanized with an overdose of sodium pentobarbital (1 ml/kg i.p.;Virbac, Australia). Once the rats were deeply anaesthetised a cardiac blood sample was taken for plasma valproate analysis via an Abbott TDX system (Abbott Diagnostics, North America). The brains were then removed, embedded in OCT compound and snap frozen in iso-pentane. 20 µm frozen sections underwent a rapid thionin stain (0.3%; Sigma-Aldrich, USA) to identify regions of interest (arcuate nucleus, thalamus at the level of nRT and somatosensory cortex) which were dissected using a sterile scalpel blade bilaterally from 5 sections per animal and transferred to sterile tubes containing 300 µl Trizol reagent (Invitrogen, USA) for downstream RNA extraction.

### Quantitative PCR (qPCR)

RNA was extracted from arcuate nucleus, thalamic and somatosensory cortical tissue using Trizol reagent (Invitrogen, USA) and treated with DNase I (Invitrogen) to remove any contaminating genomic DNA. The RNA pellet was washed with 75% ethanol, air dried and re-suspended with nuclease-free water. NanoDrop spectrophotometer readings (NanoDrop Technologies) determined RNA concentration and purity. 500 ng of RNA was reverse transcribed to cDNA using the Omniscript Reverse Transcription kit (QIAGEN) in the presence of an RNase inhibitor. qPCR was performed on 25 ng cDNA as described by Powell et al (2008) using catalogued gene expression assays for NPY (Assay ID Rn01410146_m1: Applied Biosystems) [41]. NPY mRNA levels were compared to mRNA levels of the housekeeping gene ribosomal 18S RNA using a catalogued gene expression assay for this gene (Assay ID Hs99999901_S1; Applied Biosystems). Analysis was performed using the ΔΔCT method [42].

### Statistical Analyses

All statistical analyses were performed using PRISM (GraphPad Software Inc, USA). Data not normally distributed (D’Agostino & Pearson omnibus normality test) were analysed using a two-tailed non-parametric Mann Whitney U test. p<0.05 was considered statistically significant.

## Results

Valproate was well tolerated by the animals with no marked behavioural changes observed over the course of the study. Five rats displayed less than 1% time in seizure activity during the baseline recordings and therefore were excluded from the seizure suppression, mRNA and food intake analysis, giving n = 6 for each treatment arm. Average valproate levels in cardiac blood taken at the completion of the study were 311.3±20.8 µg.ml^−1^ (n = 6) in valproate treated rats. Valproate levels measured in two saline treated rats were below the detection limit (<5 µg.ml^−1^).

### Valproate Suppresses Seizures in GAERS

**Figure 1:**
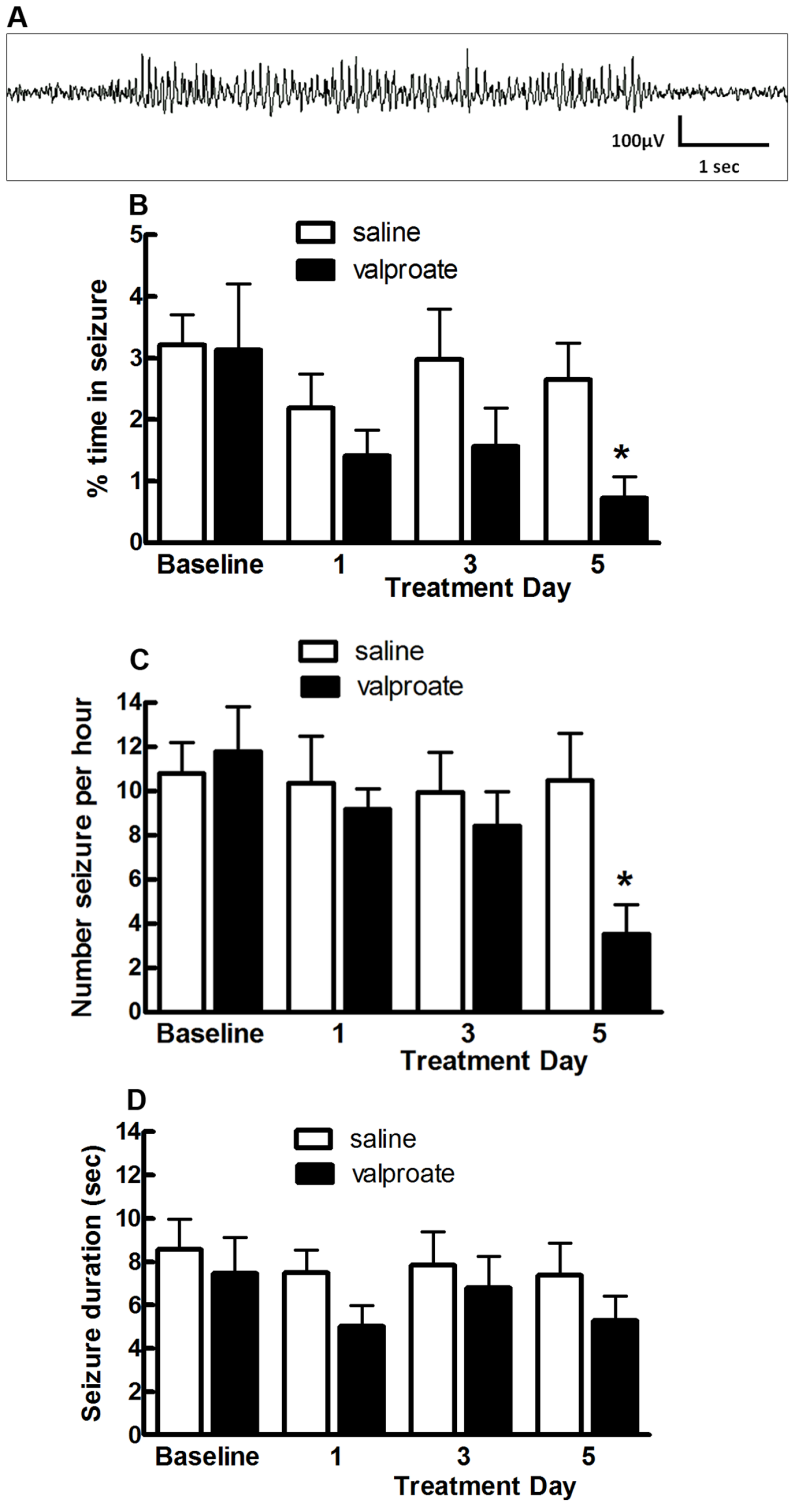
Five days of valproate treatment suppresses seizures in GAERS. (A) Representative EEG trace from a GAERS rat showing the characteristic spike-and-wave discharge of an absence seizure. GAERS were given valproate (black bars n = 6; 42 mg.kg-1 hr-1) or saline (white bars n = 6) continuously for 5 days. 24 hour EEG data were collected and the percent of time spent in seizure activity (B), number of seizures per hour (C) and seizure duration (D) was quantified for each animal. A two-tailed Mann Whitney U test at each time point shows that the percentage of time spent in seizure activity (B) and the number of seizures per hour (C) was significantly reduced only after five days of treatment. Data shown as mean±SEM *p<0.05 valproate treatment compared to saline control.

A representative EEG trace from a GAERS showing the characteristic SWD is shown in Figure 1A. GAERS were treated with a continuous infusion of either valproate (n = 6) (42 mg.kg^−1^.hr^−1^) or saline (n = 6) for 5 days. The percentage of time spent in seizure activity at baseline was not significantly different between GAERS receiving valproate treatment compared to GAERS receiving saline treatment (Fig. 1B). One day or three days of continuous valproate infusion had no significant effect on the percentage of time spent in seizure activity, however after five days of valproate treatment a significant reduction in the percentage of time spent in seizure activity was observed when compared to GAERS receiving saline treatment (Fig. 1B, p<0.05). A similar result was also observed when comparing the number of seizures per hour. Only after five days of continuous valproate treatment was a significant reduction in the number of seizures per hour observed compared to saline treated GAERS (Fig. 1C, p<0.05). Additionally, there was no significant difference in seizure duration between valproate and saline treated GAERS on each treatment day (Fig. 1D, p>0.05).

### Valproate Treatment had No Effect on Food Intake

**Figure 2:**
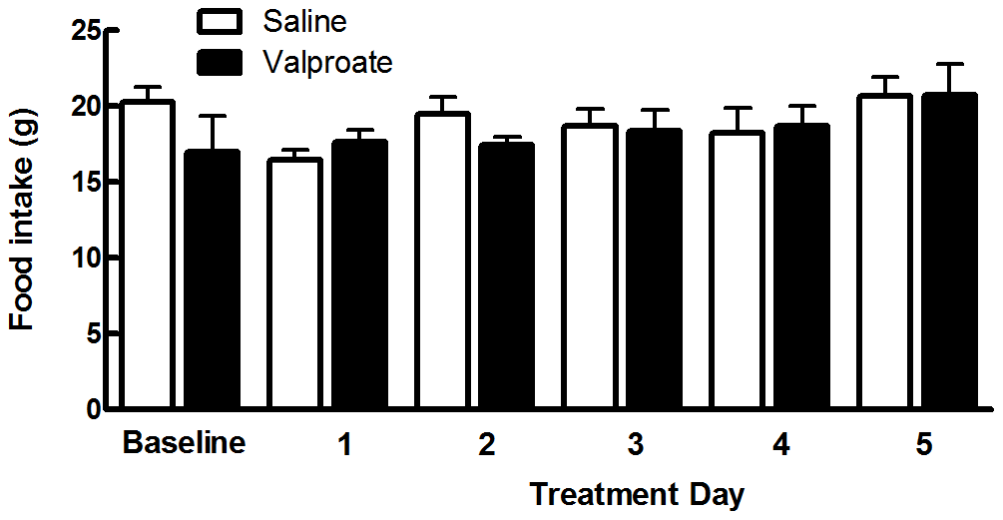
Valproate does not affect food intake in GAERS. Food intake in saline (white bars n = 6) and valproate (black bars n = 6) treated GAERS at baseline and over the 5 day treatment period. There was no significant difference in food intake between GAERS receiving valproate compared to GAERS receiving saline treatment on each day of treatment (p>0.05, two-tailed Mann Whitney U test). Data shown as mean±SEM.

Rat food was weighed at the same time every day to allow 24 hr intake to be calculated. There was no significant difference in the amount of food eaten at baseline and on each of the 5 days of treatment in GAERS receiving valproate (n = 6) compared to GAERS receiving saline (n = 6) (Fig. 2, p>0.05). On average, over the 5 day treatment period GAERS receiving valproate consumed 17.98±0.72 g of standard rat chow compared to 18.93±0.86 g for GAERS receiving saline.

### NPY mRNA Expression is Increased in the Thalamus of GAERS after Valproate Treatment

**Figure 3:**
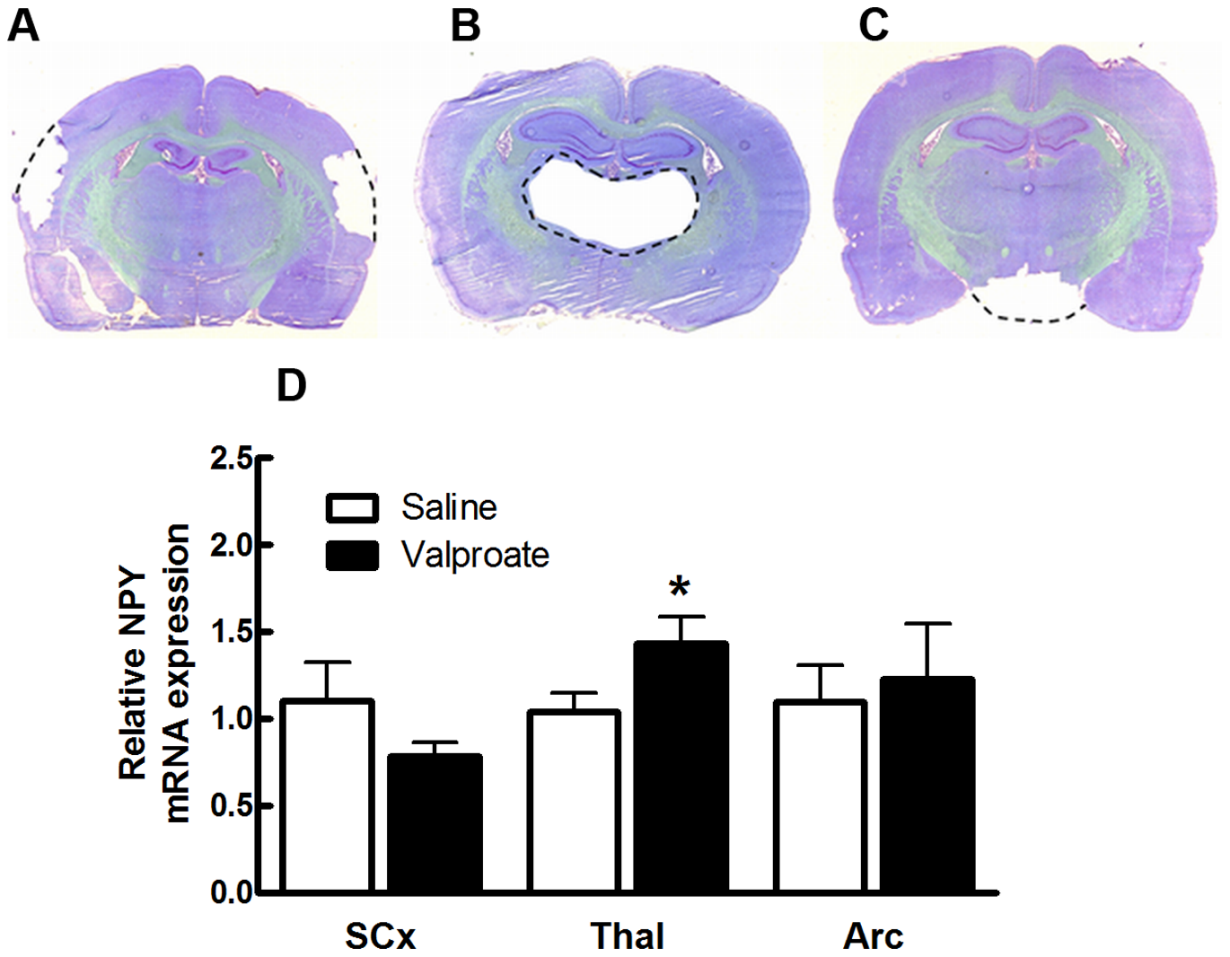
NPY mRNA expression is increased in the thalamus of valproate treated GAERS. Representative coronal pictures of dissected tissue from SCx (A); Thal (B); Arc (C). (D) NPY mRNA expression (relative to 18S mRNA expression) was significantly increased in the thalamus of valproate treated GAERS (black bars n = 6) compared to saline treated GAERS (white bars n = 6). Data shown as mean±SEM. *p<0.05 compared to saline controls (two tailed Mann Whitney U test). Thal - thalamus; SCx - Somatosensory cortex; Arc -arcuate nucleus.

NPY mRNA expression was measured from tissue dissected off coronally cut brain sections. Tissue was dissected and collected from the somatosensory cortex, thalamus and arcuate nucleus and an example of the sections with the tissue removed can be seen in Fig 3A–C respectively. GAERS receiving continuous valproate treatment for 5 days (n = 6) showed a significant increase (38%) in NPY mRNA expression in the thalamus compared to saline treatment (n = 6) (Fig. 3D, p<0.05). There was no significant difference in NPY mRNA expression in the arcuate nucleus or the somatosensory cortex of valproate treated GAERS compared to saline treatment (Fig 3D).

## Discussion

This study is the first to investigate whether NPY mRNA expression is up-regulated after valproate treatment in an animal model of genetic generalised epilepsy. We demonstrated that long-term valproate treatment suppressed seizures in GAERS and this was associated with a significant increase in NPY mRNA expression in the thalamus. These results provide support for the proposition that NPY may play a role in the mechanism by which valproate acts to suppress absence seizures.

Clinical studies have shown that maximal seizure control builds up after several weeks of valproate treatment [43]. Consistent with this, here we found that the degree of seizure suppression in GAERS only reached significance after five days of valproate treatment (Fig. 1). This is in line with a previous study from our group showing significant seizure suppression following 3 days of continuous valproate treatment in GAERS [38]. A previous study found that treating non-epileptic Sprague Dawley rats with valproate for four days resulted in a significant increase in NPY expression in the nRT and hippocampus [2]. Our current study has extended these findings to demonstrate that the increase in NPY expression in the thalamus is associated with seizure suppression. The magnitude of the increase in NPY mRNA expression in the thalamus in the previous study (30–50%) was similar to that observed in our study in valproate treated GAERS (38%). These combined data is consistent with the hypothesis that valproate causes region specific increases in NPY expression. Brill and colleagues showed that NPY expression was increased in the thalamus after four days of valproate treatment, but not after the administration of a single dose [2]. This suggests that long term treatment with valproate is required for the resultant increase in NPY expression.

Given the wide spectrum of anticonvulsant efficacy of valproate against different seizure types, it has been suggested that valproate acts through a combination of several different mechanisms. Valproate’s effect on the GABAergic system and GABA synthesis is well documented [44]. Valproate has been shown to increase GABA levels in the rodent brain [45], [46], in healthy people and epileptic patients [47], [48], [49], [50]. NPY is co-localised with GABA in neurons throughout the nRT and is released from neurons when sustained high frequency bursting, such as that seen in absence seizures [51]. Recurrent inhibitory actions of both GABA [52] and NPY [51] within the nRT have been demonstrated to desynchronize thalamic activity during high frequency bursting, reducing seizure propagation. This suggests a possible additive effect between these two transmitters.

The molecular mechanisms by which long-term valproate treatment increases NPY expression in the thalamus is likely to involve epigenetic mechanisms. Valproate is known to have inhibitory effects on histone deacetylase (HDAC) [53], [54], [55], [56], thus removing transcriptional repression. While the effect of HDAC activity on NPY expression in the thalamus is not known to date, studies have shown valproate alters the expression of several genes involved with neuronal survival/death such as, brain-derived neurotrophic factor (BDNF), glial cell-derived neurotrophic factor and hypoxia-inducing factor-1alpha [57], [58], [59], [60]. BDNF is also known to be a transcriptional marker for NPY up-regulation providing further evidence for our hypothesis [61], [62].

Weight gain is a common side effect in epilepsy patients treated with valproate [63], [64], [65]. NPY in the hypothalamus plays an important role in stimulating food intake and promoting weight gain [66], [67]. Although no significant effects on food intake were observed throughout the five day valproate treatment period, there was a trend for an increase in NPY mRNA expression in the arcuate nucleus of the hypothalamus, however this did not attain statistical significance. In humans treated with valproate, weight gain occurs with long-term use (months to years) and it is possible that the window of treatment used here was not sufficient to see an effect of valproate on food intake and weight gain in GAERS.

In conclusion, this study showed that long-term valproate treatment increased NPY mRNA expression in the thalamus and decreased seizures in a genetic rat model of absence epilepsy. These results provide support for the hypothesis that up-regulation of NPY expression plays a role in the anti-seizure effects of long-term valproate treatment.
